# A Novel Role for the Small Molecule Cinnamaldehyde in Protecting Against *P. gingivalis*–Induced Endothelial Dysfunction in Mice: Involvement of PPARγ/Akt/eNOS and Nrf2/ARE Signaling

**DOI:** 10.3390/antiox15020243

**Published:** 2026-02-12

**Authors:** Chethan Sampath, Bhavyasri Gaddam, Aaliyah C. Gray, Sasanka S. Chukkapalli, Pandu R. Gangula

**Affiliations:** 1 Department of ODS & Research, School of Dentistry, Meharry Medical College, Nashville, TN-37208, USA; csampath@mmc.edu (C.S.); bhavyasri.gaddam@mmc.edu (B.G.); agray21@mmc.edu (A.C.G.); 2 Department of Biomedical Engineering, Texas A&M University, College Station, TX-77843, USA; schukkapalli@tamu.edu

**Keywords:** *Porphyromonas gingivalis*, metabolic syndrome, high-fat diet, nuclear factor erythroid 2-related factor 2 (Nrf2), antioxidants, inflammation, cytokines, endothelial dysfunction

## Abstract

Background: Cardiovascular disease (CVD) remains the leading global cause of mortality, with endothelial dysfunction as an early driver of pathology. Periodontal disease (PD) and its pathogen *Porphyromonas gingivalis* (*Pg*) are increasingly associated with metabolic disturbances and vascular injury, yet the combined impact of microbial and dietary stressors has not been mechanistically defined. Methods: In this 24-week study, mice were subjected to chronic *Pg* infection with or without a high-fat diet (HFD). Metabolic profiling, cytokine analyses, molecular signaling assessments, and ex vivo vascular reactivity studies were performed to evaluate systemic and vascular outcomes. Results: *Pg* infection induced metabolic alterations and vascular inflammation, while HFD alone caused obesity, insulin resistance, dyslipidemia, and impaired endothelial relaxation. Combined *Pg* infection and HFD produced the most severe phenotype, with synergistically elevated cytokines, heightened TLR4/NF-κB activation, marked suppression of PPARγ and Nrf2 signaling, reduced eNOS expression, and diminished nitric oxide bioavailability. Cinnamaldehyde (CNM) supplementation improved metabolic indices, reduced inflammatory cytokines, restored PPARγ and Nrf2 activation, enhanced Akt-mediated eNOS phosphorylation, and normalized endothelial-dependent vasorelaxation. Conclusions: *Pg* infection and HFD act as synergistic metabolic and vascular stressors that accelerate endothelial dysfunction through coordinated disruption of PPARγ/Akt/eNOS and Nrf2 pathways, while CNM provides substantial protective effects.

## 1. Introduction

Periodontal disease (PD) is a consequence of chronic gingival inflammation, which ultimately leads to destruction of the tooth-supporting tissues characterized by increased periodontal pocket depth, gingival bleeding, and clinical attachment loss [1]. The progression of PD is associated with the establishment of a dysbiosis characterized by the predominance of anaerobic species, including *Porphyromonas gingivalis* (*Pg*), and an imbalanced host-immune response inducing periodontal tissue destruction [2]. Accumulating evidence for the past several years indicates that chronic PD contributes to the onset and/or progression of systemic inflammatory diseases such as cardiovascular diseases, diabetes, obesity, and several other diseases [3,4,5,6,7,8]. The prevalence of severe periodontitis is higher in diabetic patients compared with non-diabetic individuals (58% vs. 38%) with similar local irritation, including greater loss of attachment, greater alveolar bone loss, increased bleeding on probing, and increased tooth mobility resulting in tooth loss [9,10]. In addition, it has been reported that several factors, including increased oxidative stress, increased coagulability, endothelial dysfunction, and autonomic neuropathy, are often present in patients with diabetes, which may directly contribute to the development of CVD [11].

Endothelial dysfunction is a critical early event in the pathogenesis of cardiovascular disease and serves as an independent predictor of adverse cardiovascular outcomes [12,13]. A central feature of endothelial dysfunction is the loss of nitric oxide (NO) bioavailability and impaired NO-mediated vasodilation, often driven by endothelial nitric oxide synthase (eNOS) uncoupling and increased production of reactive oxygen species (ROS) [14]. Mesenteric arteries, which are key resistance vessels supplying the intestine, play an important role in regulating peripheral vascular tone and blood pressure. A major regulator of eNOS activity is tetrahydrobiopterin (BH_4_), an essential redox cofactor required for proper eNOS coupling and NO production [12]. BH_4_ deficiency or impaired regeneration leads to eNOS uncoupling, decreased NO bioavailability, and increased superoxide formation, thereby driving oxidative stress and vascular inflammation [12]. Recent evidence suggests that chronic inflammatory conditions, including periodontal infection, may disrupt BH_4_ synthesis and recycling pathways, particularly through alterations in GTP cyclohydrolase-I (GCH-I) and dihydrofolate reductase (DHFR), potentially linking periodontitis to systemic endothelial impairment [13]. Although numerous clinical, microbiological, molecular, and epidemiological studies demonstrate an association between periodontitis and endothelial dysfunction, the precise mechanisms remain poorly understood.

Antioxidant deficiencies at both local and systemic levels have been directly linked to periodontal disease, underscoring the importance of redox balance in maintaining periodontal health [14]. One of the central regulators of cellular antioxidant defenses is the transcription factor Nrf2 (nuclear factor erythroid 2–related factor 2), encoded by the Nfe2l2 gene, which regulates the expression of Phase II antioxidant and detoxification genes that, in turn, regulate levels of ROS. Vascular oxidative stress is a leading cause in the development of cardiovascular diseases [15]. Elevated oxidative stress leads to vasoconstriction, vascular remodeling, inflammation, and fibrosis [16]. Insufficient cellular protection against oxidative stress has been described as a major culprit for the development of vascular diseases. In addition, vascular oxidative stress promotes systemic inflammation via immune activation [17]. Natural antioxidants protect against tissue damage caused by free radicals and play a critical role in maintaining overall health by supporting cellular defense mechanisms against oxidative stress [18]. Cinnamaldehyde (CNM; essential oil), a key bioactive compound in cinnamon, exhibited antioxidant activity via activating Nrf2, which has been shown to have a cardiovascular protective effect [19]. However, its role in endothelial dysfunction induced by a high-fat diet and *P. gingivalis* infection remains unknown. We hypothesize that chronic periodontal infection disrupts endothelial homeostasis by impairing the BH_4_-dependent eNOS coupling and Nrf2-mediated antioxidant signaling, resulting in heightened vascular inflammation. Accordingly, this study evaluates the pharmacological effects of cinnamaldehyde (CNM) on blood pressure regulation, vascular tension, and the molecular mechanisms governing eNOS function, redox balance, and inflammatory signaling.

## 2. Materials and Methods

### 2.1. Experimental Design

All in vivo studies were conducted under an animal use protocol approved by the Institutional Animal Care and Use Committee (IACUC; protocol ID #17-09-764, approved 3 May 2018) at Meharry Medical College and adhered to the National Institutes of Health Guide for the Care and Use of Laboratory Animals. Female C57BL/6J mice (8-9 weeks of age) were obtained from The Jackson Laboratory (Bar Harbor, ME, USA) and were specific pathogen–free at study initiation. Animals were housed in groups of four per cage in the institutional vivarium under controlled environmental conditions, including a 12 h light/dark cycle, with unrestricted access to food and water. Environmental enrichment was provided, and animals were monitored daily for health and welfare. The experimental unit was the individual animal. The high-fat diet consisted of a modified diet-induced obesity (DIO) formulation (5SSV; Teklad Global 2018, Teklad Diets, Madison, WI, USA), as used in our previous studies [20,21]. This diet provides approximately 35% fat, 20% protein, and 45% carbohydrate by weight, corresponding to ~70% of total caloric intake from fat, ~20% from protein, and ~10% from carbohydrate. The normal diet consisted of standard laboratory chow (TestDiet, St. Louis, MO, USA), formulated to provide approximately 4–6% fat, 20% protein, and 70–75% carbohydrate by weight, corresponding to ~10–12% of total caloric intake from fat, consistent with our prior reports [20,21].

### 2.2. Mouse Infection

The mice were randomly distributed into seven groups (n = 8/group) based on the type of diet and treatment: Sham + standard chow (ND), sham + infection on ND, sham + infection + CNM on ND, sham + HFD, HFD + CNM, HFD + infection, and HFD + infection + CNM. CNM was prepared in mineral oil (carrier solution) and administered at a dose of 50 mg/kg, three times per week via intraperitoneal injection. The dose of cinnamaldehyde (CNM; 50 mg/kg) used in the present study was selected based on our earlier published work as well as previous studies in the literature demonstrating the biological efficacy and tolerability of CNM in murine models [22]. In our prior study, this dose was shown to effectively modulate redox signaling and vascular function without evidence of systemic toxicity [23]. After group allocation, all mice received sulfamethoxazole (0.87 mg/mL) and trimethoprim (0.17 mg/mL) in their drinking water daily for 10 days. The antibiotics were purchased from Allivet (Miami Lakes, FL, USA). Following this antibiotic treatment, the oral cavity of each mouse was rinsed with 0.12% chlorhexidine gluconate (Peridex; 3M ESPE Dental Products, St. Paul, MN, USA) once daily for 3 consecutive days to suppress endogenous murine flora and facilitate subsequent colonization by human periodontal bacteria [24,25]. *P. gingivalis* (strain W83) was cultured in our laboratory as previously described [26]. Briefly, *P. gingivalis* was inoculated into Tryptic Soy Agar (TSA) broth supplemented with hemin and menadione and grown to mid-log phase (OD_600_ ≈ 0.8) prior to preparation of the microbial inoculum. *P. gingivalis*, microbial inoculum (1 × 10^9^ bacteria per mL) was administered orally by gavage to the infection group (n = 8) for four consecutive days, every other week, for a total of 24 weeks to mimic chronic exposure of PD. Control uninfected mice (n = 8) were inoculated with sterile 2% carboxymethyl cellulose (CMC) because it matches the viscosity and physical properties of the bacterial suspension used for *P. gingivalis* administration, thereby controlling for mechanical and procedural effects associated with oral inoculation. Blood and tissues were harvested following euthanasia in accordance with institutional and NIH guidelines using CO_2_ inhalation at 24 weeks.

### 2.3. Evaluation of Glucose Regulation and Insulin Sensitivity

To assess metabolic changes, fasting blood glucose levels were measured every other week throughout the study using standard procedures [27]. An intraperitoneal glucose tolerance test (IPGTT) was conducted at week 22, followed by an insulin tolerance test (ITT) at week 23 [28]. Blood glucose data were reported as absolute concentrations.

### 2.4. Serum Analysis

Circulating insulin concentrations and lipid parameters were quantified using commercially available ELISA kits (Crystal Chem, Elk Grove Village, IL, USA) in accordance with the manufacturer’s instructions [29]. Serum nitrite, used as an index of nitric oxide metabolism, was measured using a colorimetric assay kit following the supplier’s protocol (BioVision, Milpitas, CA, USA) [29]. Insulin sensitivity was estimated using the quantitative insulin sensitivity check index (QUICKI), calculated as the reciprocal of the sum of the logarithms of fasting plasma insulin (FPI, µIU/mL) and fasting plasma glucose (FPG, mg/dL): QUICKI = 1/[log(FPI) + log(FPG)] [30]. Inflammatory biomarker serum amyloid A (SAA), an acute-phase reactant, was quantified in serum samples using a Mouse Serum Amyloid A ELISA kit (Kamiya Biomedical Company, Seattle, WA, USA), according to the manufacturer’s instructions [31]. Circulating levels of tumor necrosis factor-α (TNF-α), interleukin-1β (IL-1β), interleukin-6 (IL-6), and insulin-like growth factor-1 (IGF-1) were quantified in serum using commercially available mouse cytokine ELISA kits (Signosis, Santa Clara, CA, USA) according to the manufacturer’s instructions.

### 2.5. Blood Pressure Measurements

Systolic and diastolic blood pressure were obtained in awake mice using a tail cuff non-invasive blood pressure system (CODA; Kent Scientific Corporation, Torrington, CA, USA). A minimum of five measurements was obtained from each mouse [32]. Systolic blood pressure (SBP) recordings were obtained at a consistent time each day by the same trained investigator. To minimize stress-related variability, mice underwent a three-day acclimation period to the SBP procedure, and data collected during this phase were excluded from analysis. Baseline SBP was calculated as the mean value derived from measurements obtained over the following three consecutive days. For each recording session, animals were subjected to ten preliminary acclimation cycles, followed by fifteen measurement cycles; values from the measurement cycles were averaged to generate the daily SBP for each mouse. SBP values remained stable across the three baseline days, indicating that additional acclimation was not required.

### 2.6. Tissue Relaxation Response Measurement

Following euthanization, its vascular supply was excised and immediately placed in ice-cold physiological salt solution (PSS). The PSS consisted of (in mM): 114 NaCl, 4.7 KCl, 1.15 KH_2_PO_4_, 1.10 Na_2_HPO_4_, 1.18 MgSO_4_·7H_2_O, 15 NaHCO_3_, 1.15 CaCl_2_, and 5.0 glucose [33]. Secondary-order branches of the mesenteric artery were carefully dissected free of surrounding adipose and connective tissue. Vessel segments (~2 mm in length) were mounted on a wire myograph (Multi Myograph System-620M, DMT-USA, Inc., Ann Arbor, MI, USA) using tungsten wires (40 µm) and equilibrated for 15 min in PSS maintained at 37 °C, pH 7.4, and continuously aerated with 95% air and 5% CO_2_. Arterial segments were normalized to an internal diameter equivalent to 200–225 µm and allowed to equilibrate for an additional 15 min. Contractile responsiveness was assessed by repeated exposure to norepinephrine (NE; 5 µM) until stable contractions were achieved. Vasorelaxation responses were assessed by applying cumulative concentrations of adrenomedullin (AM; 10^−9^ to 3 × 10^−7^ M) to arterial segments that had been precontracted with norepinephrine at the concentration producing approximately 70% of maximal contraction (ED_70_), which was determined individually for each vessel. Experiments were conducted using vessels with intact endothelium unless otherwise specified. Endothelial integrity was verified by the ability of acetylcholine (3 µM) to induce greater than 80% relaxation of NE-precontracted arteries. For endothelium-denuded preparations, the luminal surface was gently rubbed with a 1 µm tungsten wire; successful removal was confirmed by the absence of acetylcholine-induced relaxation.

### 2.7. Immunoblot Analysis

Aortic and mesenteric tissues were rapidly frozen and later disrupted by ultrasonic homogenization in radioimmunoprecipitation lysis buffer supplemented with a protease inhibitor cocktail (Thermo Scientific, Rockford, IL, USA). Clarified lysates were assessed for total protein abundance using a bicinchoninic acid–based colorimetric assay [34]. For immunoblotting, 40 µg of protein from each sample was electrophoretically resolved on either 6% or 12% sodium dodecyl sulfate–polyacrylamide gels and subsequently transferred to nitrocellulose membranes by wet electroblotting. Following transfer, membranes were incubated in blocking solution containing 5% nonfat dry milk for 1 h at room temperature and then probed with primary antibodies recognizing PPARγ (sc-7273), GSK3β (sc-377213), Keap1 (sc-515432), and NQO1 (sc-376023, Santa Cruz Biotechnology, Dallas, TX, USA), AKT (9272) and phosphorylated AKT (9271, Cell Signaling Technology, Danvers, MA, USA), or endothelial nitric oxide synthase (eNOS; ab19956; Abcam, Cambridge, MA, USA). Primary antibodies were applied at dilutions ranging from 1:500 to 1:1000 in accordance with supplier specifications. Species-appropriate horseradish peroxidase–linked secondary antibodies (1:10,000; Sigma-Aldrich, St. Louis, MO, USA) were used for signal amplification. Immunoreactive proteins were detected using a chemiluminescent substrate system (Amersham Pharmacia Biotech, Piscataway, NJ, USA) following the manufacturer’s protocol. To confirm uniform protein loading, membranes were stripped and reprobed with an antibody against β-actin (A1978, Sigma-Aldrich, St. Louis, MO, USA). Digital images of the blots were acquired, and band intensities were quantified by densitometric analysis using ImageQuant LAS 500 software (GE Healthcare, Pittsburgh, PA, USA).

### 2.8. Quantification of mRNA Expression by Real-Time PCR

qRT-PCR was conducted on aorta and mesenteric artery tissue collected from each experimental group and rapidly snap-frozen in liquid nitrogen. Total RNA was isolated using the TRIzol reagent (Thermo Fisher Scientific, Waltham, MA, USA) following the manufacturer’s instructions. RNA integrity was verified with a NanoDrop spectrophotometer (Thermo Fisher Scientific, Waltham, MA, USA), and RNA concentration was determined using an Agilent 2100 Bioanalyzer (Agilent Technologies, Houston, TX, USA). To prevent genomic DNA contamination, samples were treated with RNase-free DNase (Invitrogen, Carlsbad, CA, USA). One microgram of DNase-treated RNA served as the template for cDNA generation using the iScript cDNA synthesis kit (Bio-Rad, Hercules, CA, USA). Each qRT-PCR reaction contained 1 μL of the resulting cDNA and gene-specific primers listed in Table 1. Quantitative PCR amplification was carried out using a SYBR Green–based detection system (Bio-Rad, Hercules, CA, USA). Thermal cycling consisted of an initial high-temperature activation step at 95 °C for 3 min, followed by 45 amplification cycles comprising denaturation at 95 °C for 30 s and annealing/extension at 55 °C for 1 min. Relative transcript levels were calculated using the comparative Ct (2^−ΔΔCt^) approach, with β-actin used as the reference gene for normalization. All real-time PCR analyses were performed in the Molecular Core Laboratory at Meharry Medical College.

### 2.9. Protein–Protein Interaction (PPI) Network Analysis

Protein–protein interaction network analysis was performed using the GeneMANIA web-based prediction server (https://genemania.org, accessed on 17 June 2025). Genes of interest identified from experimental analyses, including *DHFR, GCH1, NFE2L2* (Nrf2), *NOS1, NOS3, TLR4, TNF*, and *PPARG*, were used as input. GeneMANIA integrates multiple publicly available datasets encompassing physical interactions, co-expression, pathway co-membership, genetic interactions, and shared protein domains. Networks were generated using default weighting parameters and visualized as functional association maps. This analysis was used to provide an integrative context for experimentally observed changes in inflammatory, antioxidant, and nitric oxide signaling pathways and was not intended to establish causality.

### 2.10. Statistics

Results are expressed as mean values with associated standard errors. Group differences were evaluated using Student’s *t*-tests or Tukey’s multiple-comparison procedure following one-way or two-way analysis of variance, as appropriate. All statistical analyses were conducted using GraphPad Prism software (version 10; GraphPad Software, San Diego, CA, USA). Statistical significance was defined as a *p* value < 0.05.

## 3. Results

### 3.1. Supplementation of CNM Prevents Body Weight Gain, Adiposity, and Hyperglycemia in Pg-Infected, Diabetic, and Diabetic + Pg Mice

Body weight was monitored throughout the 24-week experimental period, and endpoint (24-week) body mass and weight gain are summarized in Table 2. Mice maintained on a normal diet exhibited modest increases in body weight over the study period. *P. gingivalis* infection under normal dietary conditions resulted in a modest increase in body weight at the study endpoint compared with normal-diet controls. In contrast, mice fed a high-fat diet (HFD), with or without *P. gingivalis* infection, exhibited markedly elevated body weight at 24 weeks compared with normal-diet controls (Table 2). Endpoint body weight was significantly higher in HFD-fed mice (52.3 ± 2.3 g) than in control animals (28.6 ± 0.8 g; *p* < 0.05), indicating substantial diet-induced weight gain. HFD-fed mice with infection displayed comparable elevations in body mass relative to HFD alone.

Notably, supplementation with cinnamaldehyde (CNM; 50 mg/kg) significantly attenuated endpoint body weight in HFD-fed mice, including those with *P. gingivalis* infection, as evidenced by lower body weight in the HFD + CNM group (33.3 ± 1.8 g) compared with the corresponding untreated HFD groups (Table 2; *p* < 0.05). These findings indicate that CNM limits excessive body weight accumulation under conditions of metabolic stress.

At study completion, mean liver weight was modestly increased in mice infected with *P. gingivalis* under normal-diet conditions (1.4 ± 0.1 g) compared with normal-diet sham controls (1.1 ± 0.1 g; Table 2). In contrast, mice fed a high-fat diet (HFD), with or without *P. gingivalis* infection, exhibited a marked increase in liver mass, reaching 2.5 ± 0.1 g in HFD sham mice and 2.7 ± 0.1 g in HFD-infected mice (*p* < 0.05 vs. normal diet). Cinnamaldehyde supplementation significantly attenuated HFD-associated liver enlargement, reducing liver weight to 1.4 ± 0.2 g in HFD + CNM mice and 1.60 ± 0.3 g in HFD + *P. gingivalis* + CNM mice (Table 2).In contrast, dietary supplementation with cinnamaldehyde (CNM) significantly reduced hepatic mass compared with untreated HFD and HFD plus *P. gingivalis* groups (*p* < 0.05), resulting in liver weights closer to those observed in normal-diet controls (Table 2). Kidney and visceral adipose tissue weights were also increased in mice fed a high-fat diet, with or without *P. gingivalis* infection, compared with normal-diet controls (Table 2). CNM supplementation significantly attenuated these HFD-associated increases in adipose tissue mass and partially normalized kidney weight, restoring values toward those observed in normal-diet mice.

### 3.2. CNM Supplementation Attenuates Dysregulated Glucose Metabolism Induced by P. gingivalis Infection and High-Fat Diet

To evaluate whether periodontitis triggered by periodontal pathogens exacerbates susceptibility to diet-associated metabolic dysfunction, we assessed fasting glycemia, insulin sensitivity, and glucose tolerance in mice following *P. gingivalis* infection. As shown in Table 3, fasting blood glucose levels at the 24-week study endpoint are presented for all experimental groups. At the 24-week study endpoint, fasting blood glucose levels were significantly elevated in mice infected with *P. gingivalis* under normal-diet conditions (204 ± 10 mg/dL) compared with normal-diet sham controls (124 ± 10 mg/dL; Table 3). High-fat diet (HFD) feeding resulted in a more pronounced increase in fasting glucose, reaching 310 ± 24 mg/dL in HFD sham mice and 322 ± 31 mg/dL in HFD-infected mice (*p* < 0.05 vs. normal diet). Cinnamaldehyde supplementation significantly attenuated hyperglycemia in both dietary conditions, reducing fasting glucose levels to 128 ± 11 mg/dL in ND + INF + CNM mice and to 158 ± 12 mg/dL and 140 ± 12 mg/dL in HFD + CNM and HFD + INF + CNM groups, respectively (Table 3).

Fasting insulin concentrations were also increased at 24 weeks in *P. gingivalis*–infected mice (0.6 ± 0.09 ng/mL) and in HFD-fed mice (1.1 ± 0.07 ng/mL) compared with normal-diet sham controls (0.3 ± 0.03 ng/mL; Table 3). CNM supplementation normalized insulin levels in both ND and HFD groups, restoring values toward those observed in control animals. Consistent with these findings, QUICKI values were lowest in HFD and HFD plus infection groups, indicating insulin resistance, whereas CNM-treated groups exhibited higher QUICKI values, suggestive of improved insulin sensitivity relative to their corresponding untreated groups (Table 3).

### 3.3. CNM Attenuated Systemic Endothelial Risk Markers

To assess the impact of *P. gingivalis* infection on vascular health, circulating markers associated with endothelial dysfunction were examined. Infected mice displayed modest elevations in total cholesterol, while triglyceride concentrations remained comparable to those of control animals (Table 3). In addition, increases in very low-density lipoprotein (VLDL) and low-density lipoprotein (LDL) fractions were observed across infected groups, whereas high-density lipoprotein (HDL) levels were unchanged relative to controls.

In contrast, mice subjected to a 24-week high-fat diet (HFD), either alone or in combination with *P. gingivalis* infection, exhibited pronounced dyslipidemia, characterized by significantly elevated levels of VLDL, LDL, total cholesterol, triglycerides, and free fatty acids compared with the control group (Table 3). Serum amyloid A concentrations were also markedly increased under these conditions, indicating an enhanced inflammatory state. Dietary supplementation with CNM significantly improved lipid parameters and reduced amyloid levels across all experimental groups (Table 3).

Consistent with systemic inflammation and endothelial dysfunction, circulating nitrate levels, used as an index of nitric oxide bioavailability, were significantly reduced in *P. gingivalis*–infected and high-fat-diet–fed mice compared with normal-diet sham controls (9.6 ± 0.5 µM; Table 3). Nitrate levels were reduced to 5.0 ± 0.2 µM in ND + *P. gingivalis* mice and to 3.7 ± 0.5 µM and 2.8 ± 0.7 µM in HFD and HFD + *P. gingivalis* groups, respectively (*p* < 0.05). Cinnamaldehyde supplementation significantly increased nitrate levels in both dietary conditions, restoring values toward those observed in normal-diet controls (Table 3).

### 3.4. Cinnamaldehyde Promotes Anti-Inflammatory Effects in Circulation of Pg Infection and in HFD-Fed Mice

**Figure 1:**
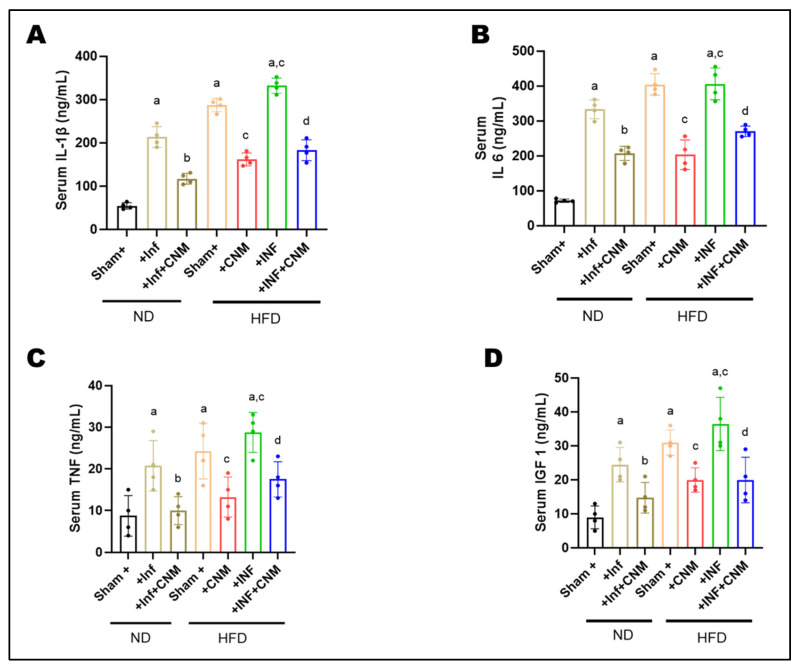
Cinnamaldehyde attenuates systemic inflammatory cytokines induced by *P. gingivalis* infection and high-fat diet (HFD) at 24 weeks. Circulating levels of inflammatory cytokines and a metabolic growth factor were measured using enzyme-linked immunosorbent assays. Serum concentrations of pro-inflammatory cytokines and metabolic growth factor were quantified by ELISA. (**A**) IL-1β, (**B**) IL-6, and (**C**) TNF-α levels were markedly elevated in *P. gingivalis*-infected (Inf) and HFD-fed mice compared with normal-diet (ND) controls (*p* < 0.05), with the highest levels observed in the combined HFD + Inf group, indicating synergistic systemic inflammation. Cinnamaldehyde (CNM) supplementation significantly reduced cytokine concentrations in Inf + CNM, HFD + CNM, and HFD + Inf + CNM groups (*p* < 0.05 vs. respective untreated groups). (**D**) Serum IGF-1 levels, which were diminished by infection and diet, were partially restored by CNM treatment (*p* < 0.05). Data are presented as mean ± SEM (n = 4 per group). ^a^ p < 0.05 vs. sham-ND; ^b^ p < 0.05 vs. *Pg*-infected ND, ^c^ p < 0.05 vs. sham-HFD and ^d^ p < 0.05 vs. *Pg*-infected +HFD. Abbreviations: ND, normal diet; Inf, *P. gingivalis* infection; HFD, high-fat diet; CNM, cinnamaldehyde; IL-1β, interleukin-1 beta; IL-6, interleukin-6; TNF-α, tumor necrosis factor-alpha; IGF-1, insulin-like growth factor 1; SEM, standard error of mean.

To examine whether improvements in metabolic outcomes were linked to changes in inflammatory status, circulating concentrations of IL-1β, IL-6, TNF-α, and IGF-1 were measured in serum. Both ND-INF and HFD groups exhibited robust elevations in IL-1β (218–287 ng/mL, Figure 1A), IL-6 (330–404 ng/mL, Figure 1B), and TNF-α (18–22 ng/mL, Figure 1C) compared with ND controls (IL-1β = 56 ng/mL, IL-6 = 72 ng/mL, TNF-α = 6 ng/mL). The combination of HFD and inflammatory challenge (HFD-INF) further exacerbated cytokine production, yielding the highest IL-6 (455 ng/mL) and TNF-α (31 ng/mL) levels, confirming synergistic enhancement of systemic inflammation (Figure 1).

Administration of CNM markedly suppressed the altered inflammatory mediators. IL-1β and IL-6 were reduced by approximately 40–50% in both ND-Inf + CNM and HFD-Inf + CNM groups relative to their untreated counterparts, while TNF-α decreased to near-control levels (11–18 ng/mL). Notably, IGF-1, which was elevated under insulin-resistant conditions (26–38 ng/mL, Figure 1D), declined toward physiological values (15-21 ng/mL) following CNM treatment, suggesting restoration of endocrine and metabolic balance (Figure 1).

### 3.5. CNM Attenuates HFD-Associated Elevations in Systolic and Diastolic Blood Pressure

**Figure 2:**
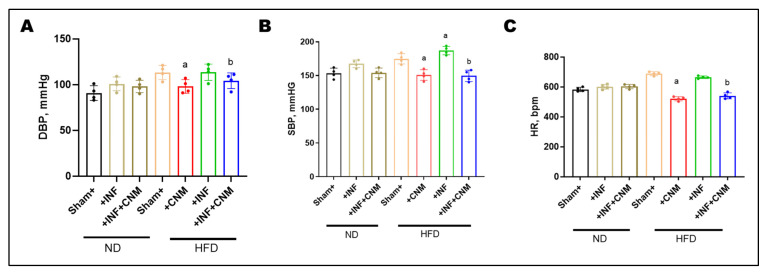
Cinnamaldehyde attenuates high-fat-diet–associated increases in blood pressure and heart rate. (**A**) Diastolic blood pressure (DBP), (**B**) systolic blood pressure (SBP), and (**C**) heart rate (HR) were measured across experimental groups. Mice fed a high-fat diet (HFD), with or without *P. gingivalis* infection (Inf), exhibited significantly increased DBP, SBP, and HR compared with normal-diet controls. Cinnamaldehyde (CNM) supplementation significantly attenuated these HFD-associated elevations and restored values toward control levels (*p* < 0.05). Data are presented as mean ± SEM (n = 4). ^a^ p < 0.05 vs. sham-HFD; ^b^ p < 0.05 vs. *Pg*-infected + HFD. Abbreviations: Inf, P*. gingivalis* infection; HFD, high-fat diet; CNM, cinnamaldehyde; DBP, diastolic blood pressure; SBP, systolic blood pressure; HR, heart rate; SEM, standard error of the mean.

To evaluate the hemodynamic impact of *P. gingivalis* infection and high-fat diet (HFD), diastolic blood pressure (DBP), systolic blood pressure (SBP), and heart rate (HR) were measured at the 24-week study endpoint (Figure 2). Normal-diet (ND) sham mice exhibited DBP and SBP values of 90.8 ± 4.0 mmHg and 153.2 ± 3.8 mmHg, respectively, with a heart rate of 583.2 ± 7.1 bpm. *P. gingivalis* infection under normal-diet conditions resulted in modest increases in DBP (100.8 ± 3.8 mmHg), SBP (167.5 ± 2.9 mmHg), and HR (601.5 ± 8.3 bpm), which did not reach statistical significance compared with ND controls. In contrast, HFD feeding produced marked elevations in all hemodynamic parameters. DBP increased to 113.2 ± 3.9 mmHg in HFD sham mice and 113.5 ± 4.5 mmHg in HFD plus *P. gingivalis*–infected mice, while SBP rose to 174.8 ± 3.8 mmHg and 187.0 ± 3.1 mmHg, respectively (*p* < 0.05 vs. ND). Heart rate was also significantly elevated under HFD conditions, reaching 688.8 ± 6.4 bpm in HFD sham mice and 664.2 ± 4.4 bpm in HFD-infected mice.

CNM supplementation significantly attenuated HFD-associated increases in blood pressure and heart rate. In HFD-fed mice, CNM reduced DBP to 98.0 ± 3.7 mmHg, SBP to 150.8 ± 4.0 mmHg, and HR to 521.0 ± 7.0 bpm. Similar improvements were observed in HFD plus *P. gingivalis*–infected mice treated with CNM, with DBP reduced to 104.2 ± 4.3 mmHg, SBP to 149.5 ± 4.2 mmHg, and HR to 540.2 ± 10.1 bpm, restoring these parameters toward values observed in ND controls (Figure 2).

### 3.6. CNM Restores Endothelium-Dependent Vasorelaxation Impaired by Pg Infection and High-Fat Diet

**Figure 3:**
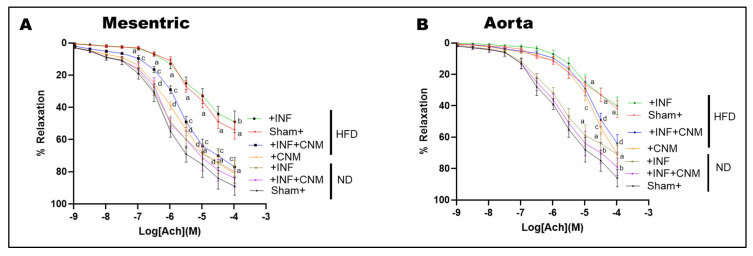
Cinnamaldehyde restores endothelial function and nitric oxide cofactor gene expression in *P. gingivalis*-infected and high-fat diet-fed mice. (**A**,**B**) Concentration response curves for acetylcholine-induced endothelium-dependent vasorelaxation in mesenteric arteries (**A**) and aortic rings (**B**). High-fat diet feeding, with or without *P. gingivalis* infection, significantly attenuated vascular relaxation compared with sham normal diet controls. Cinnamaldehyde supplementation significantly improved endothelial responsiveness under high-fat diet conditions, including in infected mice, shifting the curves toward control responses. CNM did not significantly alter vasorelaxation in normal diet mice. Data are presented as mean ± SEM (n = 4). ^a^ p < 0.05 vs. sham-ND; ^b^ p < 0.05 vs. *Pg*-infected ND, ^c^ p < 0.05 vs. sham-HFD, and ^d^ p < 0.05 vs. *Pg*-infected +HFD. Abbreviations: ND, normal diet; HFD, high-fat diet; Inf, *P. gingivalis* infection; CNM, cinnamaldehyde; ACh, acetylcholine.

To assess nitric oxide-mediated endothelial function, acetylcholine-induced vasorelaxation was evaluated in isolated mesenteric arteries and aortic rings (Figure 3). Both *P. gingivalis* infection and high-fat diet (HFD) feeding significantly impaired acetylcholine-mediated relaxation compared with sham normal-diet controls (*p* < 0.05), indicating endothelial dysfunction. The combined HFD plus infection group exhibited the greatest impairment, consistent with the additive effects of metabolic and infectious stress.

Quantitative nonlinear regression analysis of concentration–response curves revealed marked reductions in maximal vasorelaxation (Emax) and decreased acetylcholine sensitivity in HFD-fed mice. In normal-diet sham animals, Emax values were high in both mesenteric arteries and aorta (approximately 89–92%), with EC_50_ values in the low micromolar range (approximately 0.7–1.7 × 10^−6^ M; Table 4). In contrast, HFD feeding significantly reduced Emax to approximately 58% in mesenteric arteries and 60% in aorta, accompanied by rightward shifts in EC_50_ to approximately 5 × 10^−6^ M and 1.9 × 10^−5^ M, respectively, reflecting diminished endothelial responsiveness. The presence of *P. gingivalis* infection under HFD conditions further exacerbated endothelial dysfunction, reducing Emax to approximately 55% in mesenteric arteries and 45% in aorta, with EC_50_ values remaining markedly elevated (approximately 5 × 10^−6^ M in mesenteric arteries and 9 × 10^−6^ M in aorta; Table 4). CNM supplementation significantly improved endothelium-dependent vasorelaxation under HFD conditions. In HFD-fed mice treated with CNM, Emax increased to approximately 85% in mesenteric arteries and exceeded 100% in aorta, with corresponding leftward shifts in EC_50_ to approximately 1.5 × 10^−6^ M and 3.6 × 10^−5^ M, respectively (p < 0.05 vs. HFD). Similarly, in HFD plus infection mice, CNM treatment increased Emax to approximately 79% in mesenteric arteries and 90% in aorta, with partial normalization of EC_50_ values (approximately 2.1 × 10^−6^ M and 3.0 × 10^−5^ M, respectively; p < 0.05 vs. untreated HFD plus infection groups).

In contrast, CNM supplementation did not significantly alter EC_50_ or Emax in normal-diet infected mice, indicating that its protective effects on endothelial function were most evident under conditions of metabolic stress. Collectively, these findings demonstrate that CNM restores nitric oxide-dependent vasorelaxation primarily by improving maximal endothelial responsiveness and acetylcholine sensitivity in vessels compromised by HFD and chronic *P. gingivalis* infection.

### 3.7. Cinnamaldehyde Enhances BH_4_ Bioavailability to Maintain eNOS Coupling During Pg Infection and High-Fat Feeding

**Figure 4:**
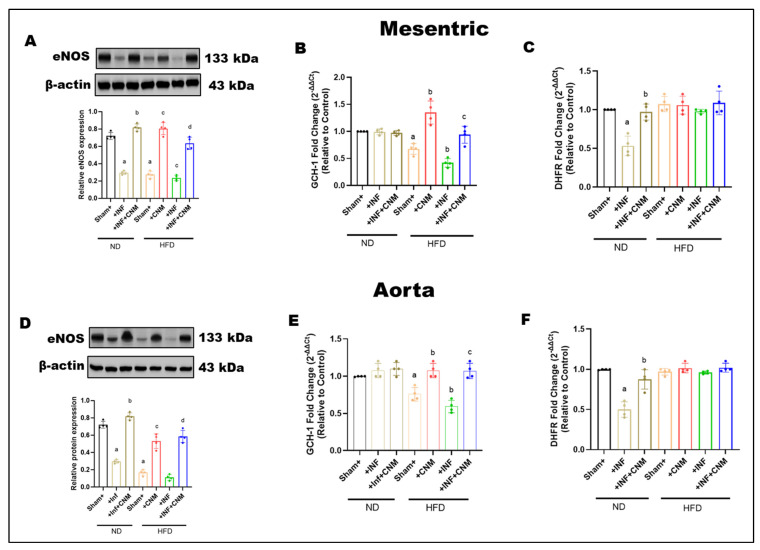
(**A**,**D**) Representative Western blots and densitometric analysis of endothelial nitric oxide synthase (eNOS, 133 kDa) in mesenteric and aortic tissues. eNOS protein levels were reduced in Inf and HFD groups, with the greatest decrease in the HFD + Inf group, whereas CNM supplementation restored eNOS expression toward ND levels (*p* < 0.05). (**B**,**C**,**E**,**F**) Quantitative PCR analysis of GCH1 and DHFR, key enzymes in tetrahydrobiopterin (BH_4_) biosynthesis, showed significant down-regulation in Inf and HFD groups (*p* < 0.05) and restoration by CNM in Inf + CNM, HFD + CNM, and HFD + Inf + CNM mice. Data are presented as mean ± SEM (n = 4). ^a^ p < 0.05 vs. sham-ND; ^b^ p < 0.05 vs. *Pg*-infected ND, ^c^ p < 0.05 vs. sham-HFD, and ^d^ p < 0.05 vs. *Pg*-infected +HFD. Abbreviations: ND, normal diet; Inf, *P. gingivalis* infection; HFD, high-fat diet; CNM, cinnamaldehyde; ACh, acetylcholine; eNOS, endothelial nitric oxide synthase; *GCH1*, GTP cyclohydrolase 1; *DHFR*, dihydrofolate reductase; BH_4_, tetrahydrobiopterin; SEM, standard error of mean. Dots shown in the bar graphs represent individual data points.

To determine whether *P. gingivalis* infection and a high-fat diet affect endothelial nitric oxide signaling and pathways that regulate tetrahydrobiopterin availability, total eNOS protein expression, and the expression of key BH_4_-related enzymes were examined in mesenteric arteries and aorta (Figure 4). In mesenteric arteries, total eNOS expression was slightly reduced in infected mice and more clearly reduced in mice fed on HFD, with or without infection, compared with normal diet controls (Figure 4A). Cinnamaldehyde supplementation partially restored eNOS expression in both HFD and HFD + infection groups. A similar pattern was observed in the aorta, where high-fat diet feeding reduced total eNOS expression regardless of infection status, and cinnamaldehyde treatment increased eNOS protein levels toward normal diet values (Figure 4D). Infection alone under normal diet conditions produced minimal changes in aortic eNOS expression.

To further examine pathways involved in BH_4_ regulation, the expression of dihydrofolate reductase (*DHFR*) and GTP cyclohydrolase I (*GCH-1*) was assessed. Infection significantly reduced dihydrofolate reductase expression in both vascular beds, while cinnamaldehyde supplementation restored its levels (Figure 4B,C). In contrast, high-fat diet feeding, with or without infection, did not significantly alter dihydrofolate reductase expression compared with normal diet controls, although cinnamaldehyde treatment maintained or slightly increased its expression.

*GCH-1* expression, the rate-limiting enzyme for de novo BH_4_ synthesis, was significantly reduced in both mesenteric arteries and aorta from mice fed a high-fat diet, including those with infection, indicating impaired BH_4_ biosynthetic capacity under metabolic stress (Figure 4E,F). CNM supplementation significantly increased *GCH-1* expression in HFD-fed mice, partially restoring levels toward those observed in normal diet controls.

HFD selectively impairs the de novo BH_4_ biosynthesis arm via reduced *GCH-1* expression, while the BH_4_ recycling pathway, as assessed by *DHFR* expression, remains largely preserved in these vascular beds. CNM supplementation helps preserve eNOS protein levels and supports pathways that regulate BH_4_ availability during metabolic and infectious stress.

### 3.8. Aortic Cytokine mRNA Expression Reflects Systemic Inflammatory Trends

**Figure 5:**
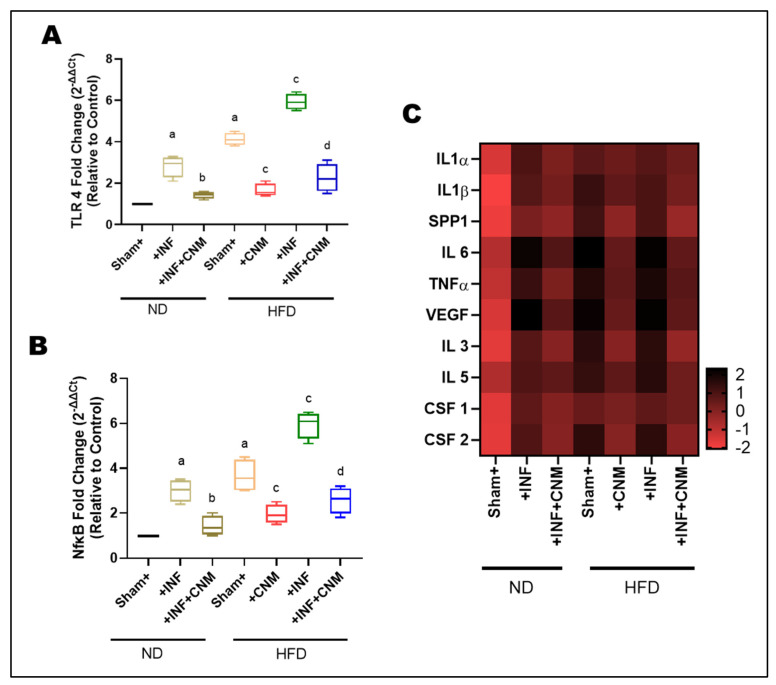
Cinnamaldehyde attenuates *TLR4/NFκB* and cytokine mRNA upregulation induced by *P. gingivalis* infection and high-fat diet. (**A**,**B**) Quantitative PCR analysis of TLR4 and NFκB gene expression in aortic tissues. Both *P. gingivalis* infection (Inf) and high-fat diet (HFD) significantly increased TLR4 and *NFκB* mRNA levels compared with sham control fed on normal diet (ND, p < 0.05). The combined HFD + Inf group exhibited the highest expression of both genes, indicating synergistic activation of inflammatory signaling. Cinnamaldehyde (CNM) supplementation markedly reduced *TLR4* and *NFκB* expression in Inf + CNM, HFD + CNM, and HFD + Inf + CNM groups (p < 0.05). (**C**) Heatmap showing differential mRNA expression of pro-inflammatory cytokines and growth factors (*IL1α*, *IL1β*, *IL6*, *TNFα*, *VEGF*, *SPP1*, *IL3*, *IL5*, *CSF1*, and *CSF2*). Expression of these genes was significantly elevated in HFD and HFD + Inf groups, whereas CNM supplementation normalized cytokine mRNA profiles toward ND levels. Data are expressed as mean ± SEM (n = 4). ^a^ p < 0.05 vs. sham-ND; ^b^ p < 0.05 vs. *Pg*-infected ND, ^c^ p < 0.05 vs. sham-HFD, and ^d^ p < 0.05 vs. *Pg*-infected +HFD. Abbreviations: ND, normal diet; Inf, P*. gingivalis* infection; HFD, high-fat diet; CNM, cinnamaldehyde; TLR4, Toll-like receptor 4; NFκB, nuclear factor kappa-light-chain-enhancer of activated B cells; IL, interleukin; TNFα, tumor necrosis factor alpha; VEGF, vascular endothelial growth factor; SPP1, secreted phosphoprotein 1; CSF, colony-stimulating factor; SEM, standard error of mean.

To investigate whether *Pg* infection and high-fat diet (HFD) promote vascular inflammation, we analyzed the expression of *TLR4* and *NFκB* in aortic tissues (Figure 5A,B). *Pg*-infection and HFD feeding significantly upregulated *TLR4* and *NF-κB* mRNA levels compared with the normal-diet (ND) control group (p < 0.05). The combined HFD + *Pg* group exhibited the highest fold increases, indicating synergistic activation of innate immune signaling. In contrast, CNM supplementation markedly suppressed both *TLR4* and *NF-κB* expression in infected and HFD-fed mice (p < 0.05), demonstrating their anti-inflammatory potential in vascular tissues.

To quantify relative transcriptional changes, fold-change values for each cytokine were calculated with respect to the ND group, normalized to 1.0. The ND+Inf and HFD groups exhibited 3-6-fold upregulation of key pro-inflammatory cytokines, including *IL-1α, IL-1β, IL-6, TNF-α*, and *SPP1* (osteopontin). These increases were accompanied by parallel elevations in *VEGF* and hematopoietic growth factors *CSF-1, CSF-2, IL-3*, and *IL-5*, indicating activation of both endothelial and myeloid inflammatory programs (Figure 5C). The HFD+Inf group displayed the highest transcript levels (6–8-fold vs. ND), highlighting synergistic amplification of vascular inflammation under combined dietary and inflammatory stress.

In contrast, CNM treatment significantly reduced cytokine expression across all classes. In ND-Inf + CNM and HFD-Inf + CNM animals, *IL-1α, IL-1β, IL-6, TNF-α*, and SPP1 expression decreased to 1.5-2.5-fold of ND levels, while *VEGF, CSF-1/2, IL-3*, and *IL-5* returned near baseline. The fold-change heatmap illustrates a coordinated normalization across multiple parameters following CNM treatment, evidenced by a shift from extreme color intensities toward intermediate hues across the spectrum, reflecting attenuation of dysregulated expression patterns (Figure 5C).

### 3.9. Modulation of PPARγ–GSK-3β/Keap1/Nrf2 Axis by Cinnamaldehyde in Vascular Tissues of Pg-Infected and HFD-Fed Mice

**Figure 6:**
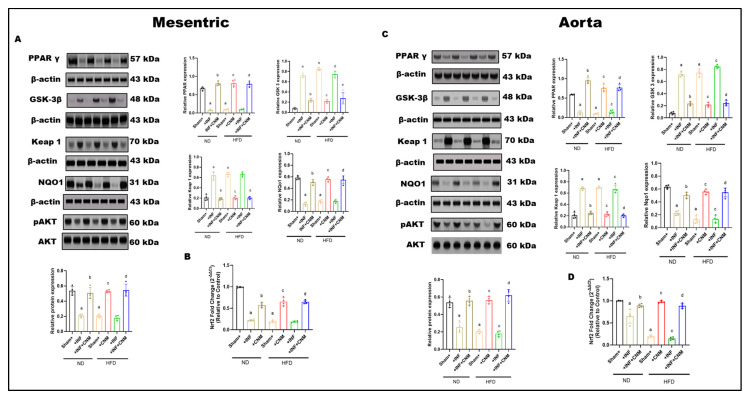
Combined effects of *P. gingivalis* infection and high-fat diet on vascular PPARγ–GSK-3β–Keap1–Nrf2–AKT signaling in mesenteric arteries and aorta. Representative Western blot images and quantitative densitometric analyses are shown for mesenteric arteries (Panel (**A**)) and aorta (Panel (**B**)). Protein expression of PPARγ (57 kDa), GSK-3β (48 kDa), Keap1 (70 kDa), NQO1 (31 kDa), total AKT (60 kDa), and phosphorylated AKT (pAKT, 60 kDa) was assessed by immunoblotting, with β-actin (43 kDa) used as a loading control. (**C**,**D**) Quantitative real-time PCR analysis of Nrf2 mRNA expression in **mesenteric arteries** (**C**) and **aorta** (**D**). Nrf2 gene expression (mRNA) was quantified separately by qRT-PCR and normalized to housekeeping genes. Animals were maintained on ND or HFD with or without *Pg* infection, as indicated. *Pg* infection under ND conditions induced moderate suppression of PPARγ and Nrf2 transcript levels, increased GSK-3β and Keap1, reduced NQO1, and attenuated AKT phosphorylation in both vascular beds. HFD alone produced a more pronounced disruption of antioxidant and survival signaling. Combined *Pg* + HFD exposure resulted in maximal suppression of Nrf2-dependent antioxidant signaling, elevated Keap1 and GSK-3β, reduced NQO1, and markedly diminished AKT phosphorylation in mesenteric arteries and aorta. Bar graphs represent mean ± SEM, expressed as relative fold change compared with sham-ND controls. ^a^ p < 0.05 vs. sham-ND; ^b^ p < 0.05 vs. *Pg*-infected ND, ^c^ p < 0.05 vs. sham-HFD and ^d^ p < 0.05 vs. *Pg*-infected +HFD.

Protein expression of PPARγ, GSK-3β, Keap1, NQO1, total AKT, and phosphorylated AKT was examined in mesenteric arteries and aortic tissues across dietary and infection conditions (Figure 6). Under normal-diet (ND) conditions, sham vessels exhibited higher PPARγ expression, lower Keap1 abundance, preserved NQO1 levels, and robust AKT phosphorylation in both vascular beds.

*P. gingivalis* infection under ND conditions was associated with significant alterations in vascular signaling. Both mesenteric and aortic tissues showed reduced PPARγ expression and increased GSK-3β and Keap1 levels compared with ND sham controls (*p* < 0.05; Figure 6A,C). These changes were accompanied by reduced NQO1 expression and decreased AKT phosphorylation, while total AKT protein levels remained unchanged.

High-fat diet (HFD) feeding in the absence of infection resulted in more pronounced changes in vascular signaling proteins. In both mesenteric arteries and aorta, HFD significantly suppressed PPARγ expression and increased GSK-3β and Keap1 levels relative to ND controls (*p* < 0.05; Figure 6A,C). NQO1 expression was concurrently reduced, and AKT phosphorylation was markedly decreased, whereas total AKT levels were not altered.

Combined exposure to *P. gingivalis* infection and HFD produced the greatest magnitude of change across all measured signaling components. In both vascular beds, the Pg + HFD group exhibited the lowest PPARγ expression, the highest GSK-3β and Keap1 levels, and markedly reduced NQO1 expression compared with all other groups (*p* < 0.05; Figure 6A,C). In parallel, Nrf2 mRNA expression was significantly reduced in mesenteric and aortic tissues in the Pg + HFD group (*p* < 0.05; Figure 6B,D). AKT phosphorylation was also lowest in this group, while total AKT protein levels remained comparable across conditions.

### 3.10. CNM Reshapes the TLR4/TNF–PPARγ/Nrf2–eNOS Signaling Network

**Figure 7:**
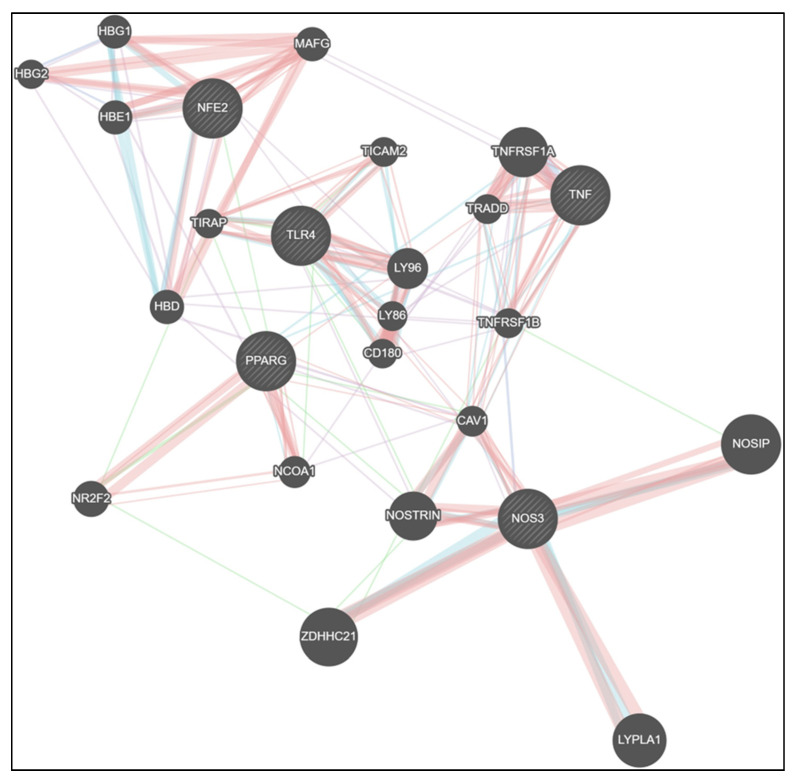
Network analysis reveals CNM-mediated rewiring of inflammatory, metabolic, and endothelial signaling pathways. GeneMANIA network visualization showing predicted and experimentally supported interactions among TLR4, TNF, PPARγ, Nrf2, and NOS3 (eNOS) gene clusters. The analysis integrates co-expression, physical interaction, pathway, and co-localization data to illustrate the interconnectivity between inflammatory (TLR4, TNF), metabolic (PPARγ), and endothelial/antioxidant (Nrf2, eNOS) regulators. CNM-responsive genes (PPARγ, Nrf2, eNOS) formed a tightly connected module inversely associated with TLR4 and TNF, suggesting CNM-driven suppression of inflammatory signaling and restoration of vascular homeostasis. Node size represents connectivity degree, and edge thickness denotes interaction strength as predicted by GeneMANIA. Abbreviations: CNM, cinnamaldehyde; TLR4, Toll-like receptor 4; TNF, tumor necrosis factor; PPARG, peroxisome proliferator-activated receptor gamma; Nrf2/NFE2, nuclear factor erythroid 2-related factor 2; NOS3, nitric oxide synthase; HBG1, hemoglobin subunit gamma 1; HBG2, hemoglobin subunit gamma 2; HBE1, hemoglobin subunit epsilon 1; MAFG, MAF bZIP transcription factor G; HBD, hemoglobin subunit delta; NR2F2, nuclear receptor subfamily 2 group F member 2; LY86, lymphocyte antigen 86; LY96, lymphocyte antigen 96; TICAM2, toll like receptor adaptor molecule 2; TIRAP, TIR domain containing adaptor protein; CD189, CD180 molecule; NCOA!, nuclear receptor coactivator 1, TNFRSF1A, Tumor Necrosis Factor Receptor Superfamily Member 1A; TRADD**, **Tumor Necrosis Factor Receptor Type 1-Associated DEATH Domain protein; CAV1, Caveolin-1; NOSTRIN, Nitric-Oxide Synthase Trafficking; ZDHHC21, ZDHHC Palmitoyltransferase 21; *LYPLA1*, Lysophospholipase 1; GeneMANIA, Gene Multiple Association Network Integration Algorithm; PPI, protein–protein interaction. Physical Interactions—Salmon/reddish; Pathway—Teal/blue-green; Co-expression—Light purple/lavender; Genetic Interactions—Green.

Protein–protein interaction (PPI) network analysis further illustrated the interconnected regulatory landscape linking inflammatory, metabolic, and redox signaling pathways modulated by CNM. The integrated network revealed strong associations between *TLR4, TNF*, and PPARγ, which were bridged to Nrf2 and endothelial nitric oxide synthase (*NOS3*) nodes, suggesting a tight crosstalk between immune and vascular homeostatic regulators. *PPARγ* and Nrf2 formed a central hub connecting to *NOSTRIN, CAV1*, and *NOSIP*, indicating that suppression of metabolic and antioxidant signaling could directly influence nitric oxide bioavailability. Conversely, CNM-responsive nodes (*PPARγ, *Nrf2*, *eNOS) were negatively correlated with *TLR4* and *TNF* clusters, consistent with experimental findings showing CNM-mediated downregulation of inflammatory regulators and restoration of vascular antioxidant defenses. Collectively, the network supports a model wherein CNM restores vascular integrity by rebalancing the *TLR4/TNF–PPARγ*/Nrf2–eNOS signaling axis disrupted by *Pg* infection and high-fat diet exposure (Figure 7).

## 4. Discussion

PD is a chronic inflammatory condition that results in alveolar bone loss and is increasingly recognized as a contributor to systemic metabolic and cardiovascular complications. Clinical evidence suggests an association between periodontal disease and metabolic as well as vascular abnormalities, including dyslipidemia, insulin resistance, endothelial dysfunction, and hypertension [35]. However, the mechanistic crosstalk between oral infection, particularly with *Pg*, and metabolic stressors such as a high-fat diet (HFD) has remained largely unexplored. Our study provides novel evidence that *Pg* + HFD synergistically worsens metabolic and vascular outcomes more than either *Pg* or HFD alone. These findings offer a translational link between clinical observations of severe cardiometabolic burden in patients with both PD and metabolic disease and the mechanistic pathways driving this interaction.

Recent epidemiological evidence, supported by experimental animal studies, has identified a link between obesity and PD [36]. Obesity is further characterized by disruptions in lipid metabolism and alterations in normal bone homeostasis. It has been reported that *P. gingivalis* infection increased body weight in male C57BL/6J mice [37]. Results from our study showed that periodontitis increased body weight, altered fasting glucose, and glucose tolerance. In addition, our animal model showed that infection with *Pg* predisposes mice to metabolic syndrome, in part by dysregulating adaptive immune responses against the pathogen. These findings are consistent with earlier clinical studies demonstrating that the presence of periodontitis exacerbates the risk and severity of metabolic disorders [38].

*Pg* infection has been proposed as one of the potential periodontal bacterial infections for the development of cardiovascular diseases [39]. We have earlier shown that polybacterial periodontal infection or *Pg* alone led to aortic atherosclerosis and modulation of lipid profiles in ApoE^null^ mice [40,41]. Vidović et al. reported that periodontal bacteria can gain access to the systemic circulation during routine oral activities, including mastication, tooth brushing, flossing, and professional dental procedures [42]. The findings further showed that viable *P. gingivalis* was identified in oral epithelial tissue as well as in the aorta, while bacterial genomic DNA was detected in multiple systemic organs [43,44]. Aortic plaque burden was significantly elevated in *Pg-infected* mice at 24 weeks (*p* < 0.01) [41]. Elevated circulating low-density lipoprotein levels are a well-recognized contributor to atherosclerotic plaque formation. Consistent with this concept, our findings indicate that prolonged *P. gingivalis* infection disrupts serum lipid homeostasis, an effect that is further amplified by high-fat diet exposure in C57BL/6J mice [37]. In both rabbits and Apoe^−^/^−^ mice, *P. gingivalis* has been shown to promote atherosclerotic plaque necrosis and exacerbate plaque instability by inducing oxidative stress-driven macrophage necroptosis [45]. While *Pg* infection alone can initiate vascular and lipid abnormalities, its impact becomes substantially more pathogenic when superimposed on underlying hyperlipidemia, where it accelerates inflammatory and metabolic dysregulation and thereby promotes the progression of coronary heart disease [46].

*P. gingivalis* possesses a distinct set of virulence determinants, including lipopolysaccharides (LPS) and gingipain proteases, which facilitate invasion of periodontal tissues and promote subsequent dissemination into the systemic circulation. The continuous infusion of LPS from *Pg* induces a chronic low-grade systemic inflammation, causing metabolic complications and disturbance in cardiac homeostasis [47]. Entry of pathogenic bacteria, including *P. gingivalis*, into the vascular endothelium triggers localized inflammatory signaling characterized by upregulation of cell adhesion molecules, Toll-like receptors, chemokines, and pro-inflammatory cytokines. [39]. Inflammatory mediators play critical roles in the initiation and progression of cardiovascular pathology. Toll-like receptors (TLRs), which serve as pattern-recognition receptors, are capable of sensing multiple virulence components expressed by *P. gingivalis*. Engagement of TLR2 by these bacterial ligands activates innate immune signaling pathways, culminating in nuclear factor-κB (NF-κB) activation. This transcriptional response promotes endothelial expression of adhesion molecules, additional TLRs, pro-inflammatory cytokines, and chemokines. Collectively, these changes are thought to drive a functional shift in endothelial cells from a quiescent, antithrombotic phenotype toward a prothrombotic and pro-inflammatory state. Activated endothelial cells subsequently facilitate recruitment of circulating monocytes, which, under conditions of elevated lipid burden—particularly oxidized low-density lipoproteins—can differentiate into foam cells and contribute to atheroma formation. This sequence of events may represent one mechanism by which *P. gingivalis* promotes vascular lesion development. In addition, *P. gingivalis* may influence cardiovascular disease indirectly through sustained periodontal infection that perpetuates systemic inflammatory signaling, including persistent elevation of mediators such as TNF-α, IL-1, interferons, IL-8, monocyte chemoattractant protein-1 (MCP-1), and C-reactive protein. These cytokines, chemokines, and acute-phase mediators could be released into the vasculature from periodontal focal *Pg* infection. Upon entering the systemic circulation, these mediators can activate vascular endothelial cells and thereby promote the progression of atherosclerotic lesions. The results from our study demonstrate the strong anti-inflammatory activity of CNM, as it effectively suppresses the upregulated pro-inflammatory cytokines induced either by *P. gingivalis* infection or by high-fat diet exposure. While protein-level analyses of additional inflammatory mediators such as NOS2 or COX-2 would have provided further mechanistic depth, the limited availability of aortic tissue following completion of vascular functional assays and gene expression analyses precluded further immunoblotting in this study. PPARγ regulates many metabolic pathways that are implicated in the pathogenesis of metabolic diseases such as metabolic syndrome, Type 2 diabetes mellitus, nonalcoholic fatty liver disease, and cardiovascular disease [48]. In obesity, inflammation is known to inhibit PPARγ expression and function. PPARγ agonists are already used for the treatment of type 2 diabetes [49]. When activated, PPARγ functions as a negative regulator of inflammatory gene expression, limiting cytokine and matrix metalloproteinase production and modulating NF-κB activation in response to oxidative stress [50]. PPARγ expression was downregulated by *Pg* and HFD, while CNM not only activates Nrf2 but also can act as a PPARγ agonist. The role of CNM as a PPARγ agonist needs to be explored further to better understand the underlying signaling cascade and use as a therapeutic compound to suppress inflammation and oxidative stress.

The phosphatidylinositol 3-kinase (PI3K)/Akt pathway serves as a central signaling axis governing diverse cellular processes, including survival, growth, differentiation, vesicle trafficking, metabolic regulation, and inflammatory responses [51]. As a principal mediator of PI3K-dependent signaling, Akt becomes activated in response to various cellular stimuli and subsequently controls the activity of downstream effectors such as Bad, caspase-9, GSK-3α/β, mTOR, and forkhead transcription factors [52]. A previous report indicates that hepatic infection with *P. gingivalis* is associated with reduced Akt activity and altered glycogen synthesis, changes that may contribute to diabetic progression [53]. The protease activity of gingipains, together with *P. gingivalis* lipopolysaccharide (Pg-LPS), has been implicated in the disruption of PI3K function and associated alterations in Akt signaling during *P. gingivalis* infection. CNM supplementation in our study has shown a potential therapeutic role in activating the vascular Akt signaling pathway. Future studies employing genetic or pharmacological inhibition of Akt or PPARγ in this combined disease model would help define pathway hierarchy and establish causal mechanisms.

Endothelial dysfunction is regarded as the first line of defense against vascular complications in cardiovascular disease [54]. Endothelial dysfunction is a key factor in the pathogenesis of hypertension and is characterized by diminished vasodilatory responses in both resistance and conduit vessels [55]. Endothelium-dependent vasorelaxation in both the aorta and mesenteric arteries is regulated by redox-sensitive mechanisms [55]. In the present study, we examined the impact of *P. gingivalis* infection and high-fat diet on oxidative stress and endothelium-dependent relaxation in the aorta; however, systemic blood pressure is primarily governed by microvascular function in resistance arteries, including the mesenteric circulation [56]. The results from our study demonstrated that endothelium-dependent relaxation in both aorta and mesenteric arteries was impaired with Pg infection and further exacerbated with HFD, whereas CNM supplementation attenuated endothelial dysfunction.

Endothelial dysfunction is a recognized consequence of cardiovascular risk exposure and represents an early event in the atherosclerotic process. Proper vascular endothelial function is largely dependent on the activity of endothelial nitric oxide synthase [57]. Endothelial nitric oxide synthase catalyzes the production of nitric oxide from L-arginine in endothelial cells and is essential for the regulation of vascular tone and maintenance of blood flow homeostasis [58]. A study by Liao et al. demonstrated that eNOS-deficient mice are hypertensive and exhibit features consistent with metabolic syndrome, such as insulin resistance, altered lipid profiles, and increased adiposity [59]. Endothelial nitric oxide synthase is constitutively expressed in endothelial cells, with its transcriptional activity modulated by cytokine signaling [58]. It is evident that activated PPARγ increases the production of NO in endothelial cells [60]. In this study, we showed that NO production was significantly lower in the *P. gingivalis*-infected group than in the control group, while eNOS expression significantly increased in the CNM-supplemented groups. Similar results were observed with CNM supplementation, enhancing eNOS expression in diabetic mice [61]. Earlier, we reported that NO levels were significantly reduced with mono or polybacterial infection, and the same was observed in HFD-fed mice [29,62,63,64].

Although CNM is widely used as a food flavoring agent and is generally considered safe for dietary use. The dose and route of administration used in this study exceed those typically achieved through normal consumption. Previous experimental studies have reported that cinnamaldehyde is well tolerated in both vertebrate and invertebrate animal models and does not exhibit detectable genotoxic activity under standard experimental conditions [65]. While our findings demonstrate clear vascular and metabolic benefits of CNM under conditions of metabolic and inflammatory stress, these results should be interpreted with appropriate caution. Notably, over the 24-week study period, CNM-treated mice did not exhibit evidence of overt systemic toxicity, including abnormal weight loss, organ dysfunction, or increased mortality. Instead, CNM supplementation was associated with improved metabolic indices, reduced inflammatory burden, and restoration of endothelial function. Nevertheless, we acknowledge that comprehensive toxicological profiling, including formal genotoxicity assessments and detailed dose–response studies, was beyond the scope of the present work. Recent reviews have emphasized that long-term safety, optimal dosing, and therapeutic windows for CNM remain incompletely defined, particularly at pharmacological doses [66,67]. Accordingly, future studies using clinically relevant routes of administration, especially oral delivery, will be essential to define safe and effective dosing strategies and to facilitate translation of these findings to human applications.

## 5. Conclusions

In conclusion, this study demonstrates that chronic oral *P. gingivalis* infection exacerbates obesity and insulin resistance and leads to more severe vascular dysfunction, particularly under high-fat diet conditions. These effects are associated with impaired nitric oxide and Nrf2-dependent antioxidant signaling, highlighting a mechanistic link between periodontal infection, metabolic stress, and vascular injury. Importantly, cinnamaldehyde supplementation attenuated vascular inflammation and oxidative stress and improved endothelial function in mice exposed to *P. gingivalis*, high-fat diet, or their combination.

Our findings indicate that the vascular protective effects of cinnamaldehyde are mediated, at least in part, through restoration of NO and Nrf2 signaling, with PPARγ emerging as an important downstream regulator of vascular homeostasis. Together, these results support a dual anti-inflammatory and antioxidant role for cinnamaldehyde in mitigating periodontal- and diabetes-associated vascular dysfunction. While further studies are required to define long-term safety, optimal dosing, and translational relevance, our data suggest that dietary bioactive compounds such as cinnamaldehyde may represent a promising adjunctive lifestyle-based strategy for reducing vascular risk in populations affected by periodontitis and metabolic disease.

## Figures and Tables

**Table 1:** Primer sequences.

Gene	NCBI RefSeq Accession No. (mRNA)	Forward Primer (5′–3′)	Reverse Primer (5′–3′)
GTPCH1 (*GCH1*)	NM_008102	AGCAAGTCCTTGGTCTCAGTAAAC	ACCGCAATCTGTTTGGTGAGGC
DHFR	NM_010049	CAGTCTCTGTTCCACTCTCTGTCCC	AAAGAGGGGCAGAATCAGGTATGGG
TLR4	NM_021297	AGCTTCTCCAATTTTTCAGAACTTC	TGAGAGGTGGTGTAAGCCATGC
NF-κB (*Nfkb1*)	NM_008689	GCTGCCAAAGAAGGACACGACA	GGCAGGCTATTGCTCATCACAG
IL-1α (*Il1a*)	NM_010554	ACGGCTGAGTTTCAGTGAGACC	CACTCTGGTAGGTGTAAGGTGC
IL-1β (*Il1b*)	NM_008361	TGGACCTTCCAGGATGAGGACA	GTTCATCTCGGAGCCTGTAGTG
SPP1 (Osteopontin)	NM_009263	GCTTGGCTTATGGACTGAGGTC	CCTTAGACTCACCGCTCTTCATG
IL-6 (*Il6*)	NM_031168	TACCACTTCACAAGTCGGAGGC	CTGCAAGTGCATCATCGTTGTTC
TNF-α (*Tnf*)	NM_013693	GGTGCCTATGTCTCAGCCTCTT	GCCATAGAACTGATGAGAGGGAG
VEGF-A (*Vegfa*)	NM_009505	CTGCTGTAACGATGAAGCCCTG	GCTGTAGGAAGCTCATCTCTCC
IL-3 (*Il3*)	NM_010556	CCTGCCTACATCTGCGAATGAC	GAGGTTAGCACTGTCTCCAGATC
IL-5 (*Il5*)	NM_010558	GATGAGGCTTCCTGTCCCTACT	TGACAGGTTTTGGAATAGCATTTCC
*CSF1* (M-CSF)	NM_007778	GCCTCCTGTTCTACAAGTGGAAG	ACTGGCAGTTCCACCTGTCTGT
*CSF2* (GM-CSF)	NM_009969	AACCTCCTGGATGACATGCCTG	AAATTGCCCCGTAGACCCTGCT
Nrf2 (*Nfe2l2*)	NM_010902	CAGCATAGAGCAGGACATGGAG	GAACAGCGGTAGTATCAGCCAG

**Table 2:** Changes in body, liver, kidney, and adipose tissue weights following 24 weeks of *P. gingivalis* infection and high-fat diet with or without cinnamaldehyde.

	ND			HFD			
	Sham +	+ Inf	+ Inf+CNM	Sham +	+ CNM	+ Inf	+Inf+CNM
Body weights (g)	28.6 ± 0.9	34.6 ± 1.4 ^a^	29.2 ± 0.8 ^b^	52.3 ± 2.0 ^a^	33.3 ± 1.8 ^c^	54.1 ± 1.7 ^c^	35.3 ± 1.6 ^d^
Liver weights (g)	1.1 ± 0.1	1.4 ± 0.1 ^a^	1.1 ± 0.1 ^b^	2.5 ± 0.1 ^a^	1.4 ± 0.2 ^c^	2.7 ± 0.1 ^c^	1.6 ± 0.3 ^d^
Liver/body weight (%)	3.74 ± 0.3	4.0 ± 0.3	3.8 ± 0.3	4.8 ± 0.3 ᵃ	4.2 ± 0.3	5.0 ± 0.3 ᵃ	4.5 ± 0.3 ᵇ
Kidney weights (g)	0.5 ± 0.03	0.5 ± 0.02	0.5 ± 0.02	0.6 ± 0.05 ^a^	0.5 ± 0.03 ^b^	0.7 ± 0.03	0.5 ± 0.04 ^c^
Adipose tissue (g)	0.3 ± 0.03	0.7 ± 0.04 ^a^	0.5 ± 0.03 ^b^	2.4 ± 0.06 ^a^	0.7 ± 0.3 ^b^	3.3 ± 0.12 ^c^	1.5 ± 0.3 ^d^

Data are presented as mean ± SEM (n = 6–8 per group). Liver-to-body weight ratios were calculated from the mean liver and body weight values shown in the table. ^a^ p < 0.05 vs. sham-ND; ^b^ p < 0.05 vs. *Pg*-infected ND, ^c^ p < 0.05 vs. sham-HFD and ^d^ p < 0.05 vs. *Pg*-infected +HFD based on one-way ANOVA with subsequent Tukey-adjusted pairwise comparisons. Abbreviations: ND = normal diet; Inf = *P. gingivalis* infection; CNM = cinnamaldehyde; HFD = high-fat diet.

**Table 3:** Effect of *P. gingivalis* infection, high-fat diet, and cinnamaldehyde supplementation at 24 wks on metabolic and serum biochemical parameters.

	ND			HFD			
	Sham+	+Inf	+Inf+CNM	Sham+	+CNM	+Inf	+Inf+CNM
Fasting blood glucose (mg/dL)	124 ± 10	204 ± 10 ^a^	128 ± 11 ^b^	310 ± 24 ^a^	158 ± 12 ^c^	322 ± 31 ^c^	140 ± 12 ^d^
Fasting insulin (ng/mL)	0.3 ± 0.03	0.6 ± 0.09 ^a^	0.3 ± 0.1 ^b^	1.1 ± 0.07 ^a^	0.6 ± 0.1 ^c^	1.1 ± 0.04 ^c^	0.7 ± 0.14 ^d^
QUICKI	0.6 ± 0.02	0.5 ± 0.05	0.6 ± 0.06	0.4 ± 0.08	0.5 ± 0.04	0.4 ± 0.06	0.5 ± 0.1
Nitrate levels (µM)	9.6 ± 0.5	5.0 ± 0.2 ^a^	7.5 ± 0.6 ^b^	3.7 ± 0.5 ^a^	5.9 ± 0.3 ^c^	2.8 ± 0.7 ^c^	4.6 ± 0.5 ^d^
SAA (ng/mL)	1.5 ± 0.3	1.9 ± 0.2 ^a^	1.6 ± 0.3 ^b^	2.6 ± 0.3 ^a^	2.0 ± 0.1 ^c^	3.0 ± 0.3 ^c^	2.5 ± 0.3 ^d^
VLDL (mg/dL)	198 ± 13	289 ± 14 ^a^	197 ± 10 ^b^	399 ± 9 ^a^	265 ± 20 ^c^	455 ± 24 ^c^	309 ± 36 ^d^
LDL (mg/dL)	115 ± 8	174 ± 27 ^a^	151 ± 12 ^b^	182 ± 9 ^a^	140 ± 10 ^c^	187 ± 8 ^c^	145 ± 18 ^d^
HDL (mg/dL)	43 ± 4	42 ± 6	42 ± 2	36 ± 3 ^a^	46 ± 4 ^b^	32 ± 3 ^c^	42 ± 4 ^d^
Total Cholesterol (mg/dL)	298 ± 10	376 ± 11 ^a^	318 ± 23 ^b^	392 ± 10 ^a^	341 ± 18 ^b^	403 ± 5 ^c^	351 ± 24 ^d^
Total triglycerides (mg/dL)	139 ± 10	173 ± 15 ^a^	138 ± 20 ^b^	295 ± 17 ^a^	255 ± 30 ^b^	412 ± 27 ^c^	341 ± 22 ^d^

Values are presented as mean ± SEM (n = 6–8 per group). ^a^ *p* < 0.05 vs. sham-ND; ^b^ p < 0.05 vs. *Pg*-infected ND, ^c^ p < 0.05 vs. sham-HFD and ^d^ p < 0.05 vs. *Pg*-infected +HFD based on one-way ANOVA with subsequent Tukey-corrected pairwise testing. Abbreviations: ND = normal diet; INF = *P. gingivalis* infection; CNM = cinnamaldehyde; HFD = high-fat diet; SAA = serum amyloid A; VLDL = very-low-density lipoprotein; LDL = low-density lipoprotein; HDL = high-density lipoprotein.

**Table 4:** Acetylcholine-induced endothelium-dependent vasorelaxation parameters in mesenteric arteries and sorta.

Group	Mesenteric Arteries		Aorta	
	Emax (%)	EC_50_ (M)	Emax (%)	EC_50_ (M)
ND sham	88.9	7.25 × 10^−7^	91.5	1.7 × 10^−6^
ND + *P. gingivalis*	80.4	7.24 × 10^−7^	74.7	1.5 × 10^−6^
ND + *P. gingivalis* + CNM	85.6	9.57 × 10^−7^	83.8	1.6 × 10^−6^
HFD sham	58.3	5.11 × 10^−6^	60.0	1.9 × 10^−5^
HFD + CNM	85.2	1.53 × 10^−6^	105.7	3.6 × 10^−5^
HFD + *P. gingivalis*	54.5	5.00 × 10^−6^	44.9	9.1 × 10^−6^
HFD + *P. gingivalis* + CNM	78.8	2.08 × 10^−6^	90.0	3.0 × 10^−5^

Emax and EC_50_ values were derived from acetylcholine concentration–response curves using four-parameter logistic nonlinear regression. Values shown represent group-level estimates obtained from mesenteric arteries and aortic rings analyzed separately. Abbreviations: ND = normal diet; CNM = cinnamaldehyde; HFD = high-fat diet; Emax: Maximum effect (maximum vasorelaxation); EC_50_: Half-maximal effective concentration; M: molar concentration; %: Percent.

## Data Availability

The original contributions presented in this study are included in the article. Further inquiries can be directed to the corresponding author.

## Abbreviations

The following abbreviations are used in this manuscript:

ACh	Acetylcholine
Akt	Protein kinase B
ANOVA	Analysis of variance
ARE	Antioxidant response element
BH_4_	Tetrahydrobiopterin
BCA	Bicinchoninic acid
CMC	Carboxymethyl cellulose
CNM	Cinnamaldehyde
CO_2_	Carbon dioxide
CSF	Colony-stimulating factor
CVD	Cardiovascular disease
DBP	Diastolic blood pressure
DHFR	Dihydrofolate reductase
ELISA	Enzyme-linked immunosorbent assay
eNOS	Endothelial nitric oxide synthase
FFA	Free fatty acids
GCH-I	GTP cyclohydrolase-I
GSK-3β	Glycogen synthase kinase-3 beta
HFD	High-fat diet
HDL	High-density lipoprotein
HR	Heart rate
IGF-1	Insulin-like growth factor-1
IL	Interleukin
INF	*Porphyromonas gingivalis* infection
IPGTT	Intraperitoneal glucose tolerance test
ITT	Insulin tolerance test
Keap1	Kelch-like ECH-associated protein 1
LDL	Low-density lipoprotein
LPS	Lipopolysaccharide
ND	Normal diet
NE	Norepinephrine
NF-κB	Nuclear factor kappa-light-chain-enhancer of activated B
NO	Nitric oxide
NOS3	Endothelial nitric oxide synthase gene
NQO1	NAD(P)H quinone dehydrogenase 1
Nrf2	Nuclear factor erythroid 2-related factor 2
PD	Periodontal disease
Pg	*Porphyromonas gingivalis*
PI3K	Phosphatidylinositol 3-kinase
PPARγ	Peroxisome proliferator-activated receptor gamma
PPI	Protein–protein interaction
PSS	Physiological salt solution
qRT-PCR	Quantitative real-time polymerase chain reaction
QUICKI	Quantitative insulin sensitivity check index
RIPA	Radioimmunoprecipitation assay
ROS	Reactive oxygen species
SAA	Serum amyloid A
SBP	Systolic blood pressure
SEM	Standard error of the mean
SPP1	Secreted phosphoprotein 1 (osteopontin)
TLR4	Toll-like receptor 4
TNF-α	Tumor necrosis factor-alpha
VEGF	Vascular endothelial growth factor
VLDL	Very low-density lipoprotein
